# Supplementary material for: Hierarchical secure key assignment scheme
Source: PLoS One. 2026 Feb 18;21(2):e0341637. doi: 10.1371/journal.pone.0341637 (PMC12915986; doi:10.1371/journal.pone.0341637)
Supplement: S1 Appendix — (HTML) [file pone.0341637.s001.html]

{
"cells": [
{
"cell\_type": "code",
"execution\_count": null,
"id": "f177ab2d-cbf6-41d0-b1e9-b33c19fccdcc",
"metadata": {
"tags": []
},
"outputs": [],
"source": [
"import time\n",
"import matplotlib.pyplot as plt\n",
"import numpy as np\n",
"from sage.all import \*\n",
"import random\n",
"# Projection function using SageMath’s vector inner product and norm\n",
"def proj(v, u):\n",
" a = (v.inner\_product(u)) / (u.norm() \*\* 2) \* u\n",
" return a\n",
"#Gram–Schmidt orthogonalization using SageMath’s vector norms and inner products\n",
"def Gram\_Schmidt(S):\n",
" n = len(S)\n",
" E = [S[0] / S[0].norm()]\n",
" for k in range(1, n):\n",
" q = S[k] - sum([proj(S[k], E[i]) for i in range(k)])\n",
" E.append(q / q.norm())\n",
" return E\n",
"#Constructing a basis using SageMath’s random vector generation and matrix rank functions\n",
"def generate\_basis(W):\n",
" new\_basis = []\n",
" while len(new\_basis) < W.dimension():\n",
" v = W.random\_element()\n",
" if v not in new\_basis and v != 0:\n",
" temp\_basis = new\_basis + [v]\n",
" if Matrix(temp\_basis).rank() == len(temp\_basis):\n",
" new\_basis.append(v)\n",
" return new\_basis\n",
"#Constructing multiple bases using SageMath’s basis construction function\n",
"def generate\_multiple\_bases(W, num\_bases):\n",
" bases = []\n",
" for \_ in range(num\_bases):\n",
" bases.append(generate\_basis(W))\n",
" return bases\n",
"#Generating random polynomials using SageMath’s polynomial ring and random polynomial generation functions\n",
"def get\_random\_func(fd):\n",
" R. = PolynomialRing(RealField(1000))\n",
" return R.random\_element(degree=fd)"
]
},
{
"cell\_type": "code",
"execution\_count": null,
"id": "1a1f9d6e-0d6d-4447-a60a-d41f869f5951",
"metadata": {
"tags": []
},
"outputs": [],
"source": [
"def check\_list(inner\_product):\n",
" epsilon = 0.00001\n",
" if len(inner\_product) == 0:\n",
" return \"Not OK\" \n",
" first\_value = inner\_product[0]\n",
" for value in inner\_product:\n",
" if abs(value - first\_value) > epsilon:\n",
" return \"Not OK\"\n",
" return \"OK\"\n",
"\n",
"def init(num\_bases):\n",
" R = RealField(20)\n",
" start\_time = time.time()\n",
" d = 10 #The dimension of E\n",
" E = VectorSpace(R, d) #SageMath's vector space construction function\n",
" n = 7 #The dimension of W\n",
" subspace = [E.random\_element() for \_ in range(n)] \n",
" W = E.span(subspace) #SageMath’s subspace construction function\n",
" #Constructing multiple bases\n",
" bases = [W.basis() for \_ in range(n)]\n",
" #Constructing orthogonal bases using Gram–Schmidt\n",
" orthogonal\_bases = [Gram\_Schmidt(basis) for basis in bases]\n",
" #Random basis selection\n",
" secret\_basis = random.choice(orthogonal\_bases)\n",
" random\_polys = get\_random\_func(10)\n",
" x\_i = np.random.choice(range(1, 1500), num\_bases, replace=False)\n",
" x\_i = list(map(ZZ, x\_i))\n",
" r\_i = [1]\n",
" r\_i += [random.randint(2, 100) for \_ in range(n - 1)]\n",
" results = [random\_polys(x) for x in x\_i]\n",
" r\_v = [r\_i[i] \* secret\_basis[i] for i in range(len(r\_i))]\n",
" final\_bases = [[res \* sb for sb in r\_v] for res in results]\n",
" v = E.random\_element()\n",
" g = E.random\_element()\n",
" projection = [sum([proj(v, i) for i in fb]) for fb in final\_bases]\n",
" inner\_product = [proj\_l.inner\_product(g) for proj\_l in projection]\n",
" end\_time = time.time()\n",
" elapsed\_time = end\_time - start\_time\n",
" print(check\_list(inner\_product))\n",
" return elapsed\_time\n",
"\n"
]
},
{
"cell\_type": "code",
"execution\_count": null,
"id": "a9b4ee32-49e2-4a8b-9a5e-968740898a86",
"metadata": {
"tags": []
},
"outputs": [
{
"name": "stdout",
"output\_type": "stream",
"text": [
"OK\n",
"OK\n",
"OK\n",
"OK\n",
"OK\n",
"OK\n",
"OK\n",
"OK\n",
"OK\n",
"OK\n"
]
}
],
"source": [
"num\_bases\_values = [100, 200, 300, 400, 500, 600, 700, 800, 900, 1000]\n",
"times\_1 = [init(num\_bases) for num\_bases in num\_bases\_values]\n",
"\n"
]
},
{
"cell\_type": "code",
"execution\_count": null,
"id": "c159e8ed-b8d3-48a2-8d18-781cfe066f0c",
"metadata": {},
"outputs": [],
"source": []
}
],
"metadata": {
"kernelspec": {
"display\_name": "SageMath 10.5",
"language": "sage",
"name": "sagemath-10.5"
},
"language\_info": {
"codemirror\_mode": {
"name": "ipython",
"version": 3
},
"file\_extension": ".py",
"mimetype": "text/x-python",
"name": "python",
"nbconvert\_exporter": "python",
"pygments\_lexer": "ipython3",
"version": "3.12.5"
}
},
"nbformat": 4,
"nbformat\_minor": 5
}
