# Supplementary material for: Hierarchical secure key assignment scheme
Source: PLoS One. 2026 Feb 18;21(2):e0341637. doi: 10.1371/journal.pone.0341637 (PMC12915986; doi:10.1371/journal.pone.0341637)
Supplement: Attachment — Submitted filename: Rebuttal letter.pdf [file pone.0341637.s003.pdf]

Point-by-Point Response

Baris Celiktas, Ibrahim Celikbilek, Sueda Guzey, Enver Ozdemir
(Corresponding author: Baris Celiktas)

December 5, 2025

Response to Reviewer #1

Comment 1: The authors presented a novel hierarchical key assignment mechanism for access control, designed to be computationally lightweight and optimized for digital environments with structured access policies.

Response 1: We thank you for your time to review our manuscript and we appreciate the recognition that the proposed scheme is computationally lightweight and suitable for structured access control environments.

Comment 2: The idea presented in this manuscript appears similar to the authors' previous IEEE Access article, which is cited as Ref. 42. The present manuscript seems to be a continuation or extension of that work.

Response 2:

We sincerely appreciate your careful review and for highlighting the point requiring clarification. The present study constitutes the pioneering work that introduces and develops the use of inner product spaces for hierarchical key access mechanisms. In parallel, the corresponding author's doctoral dissertation investigated related aspects of this topic, thereby providing complementary foundations.

An earlier version of this research, containing preliminary results, was posted on TechRxiv, and this work was duly cited in Ref.42. The contribution in Ref.42 primarily emphasizes systematic performance analysis of our proposed approach in comparison with competing methods. The findings reported therein further support the practicality and effectiveness of our framework.

Our current work is the first comprehensive study to articulate multiple perspectives on the application of inner product spaces in key access mechanisms. This includes a formal security analysis and the introduction of diverse strategies for constructing secret subspaces for groups extensions that were incorporated into the preliminary version posted on TechRxiv. To ensure transparency, we have added a dedicated paragraph (see page. . .) outlining the chronology of our studies and the progressive development of this research direction.

Comment 3: Equations are not properly numbered, and the equation style and citation formatting are inconsistent.

Response 3: We thank you for identifying these formatting inconsistencies. All equations have now been renumbered using the standard `\begin{equation}` environment, and the citation style has been unified according to the PLOS ONE formatting guidelines.

Comment 4: Figure 4 needs to be redesigned.

Response 4: We appreciate your suggestion to improve the clarity and technical structure of Figure 4. In response, the figure has been thoroughly redesigned to present a concise, step-wise illustration of the proposed hierarchical key assignment algorithm. The updated version now explicitly depicts the principal computational phases, thereby enhancing readability and technical precision, including:

- initialization of basis structures and dimensional policies,
- construction of group and user-level bases,
- generation of random public vectors and scaling constants,
- input mapping and orthogonal projection operations,
- derivation of group keys, and
- dynamic rekeying when a basis is compromised.

The revised figure provides an algorithmic, mathematically oriented workflow rather than explanatory text blocks, thereby improving clarity, technical precision, and overall readability as requested.

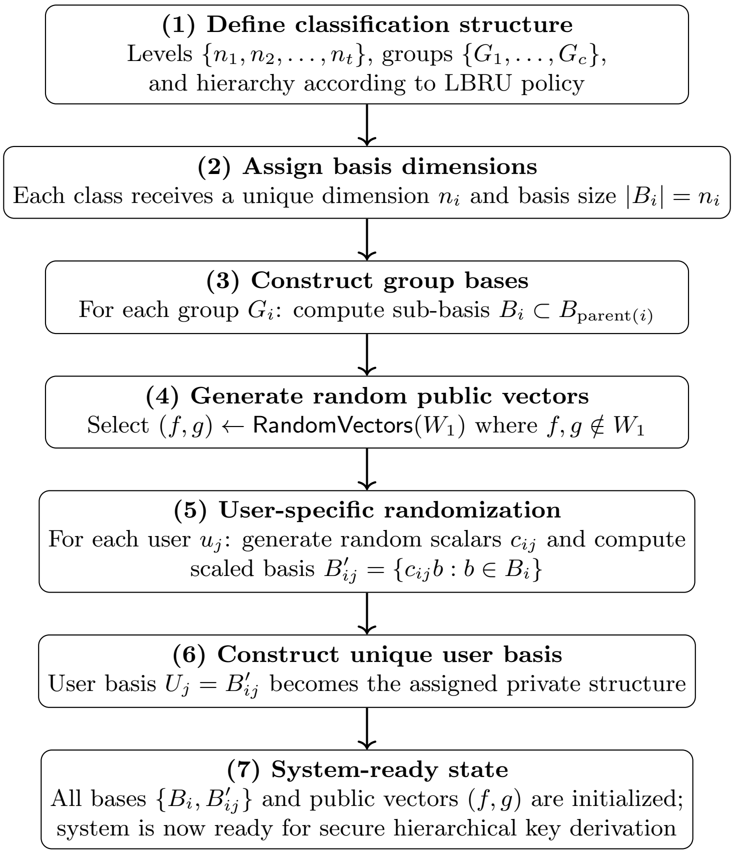

Figure 1: Steps of the system-ready phase.

Response to Reviewer #2

Comment 1: The abstract did not mention any numerical values for the metrics that demonstrate the superiority of the proposed technology.

Response 1: We thank you for your constructive suggestion. In accordance with your recommendation, we have revised the abstract to incorporate concrete numerical performance indicators that highlight the efficiency and scalability of the proposed scheme in comparison with existing alternatives. Specifically, we now include the analytical upper bound of the key derivation cost, as well as the compactness of the key material under practical deployment parameters (e.g., m=32, d=10), which results in approximately 9,920 bits per user—significantly lower than competing approaches. These additions aim to demonstrate the lightweight nature and practical applicability of the proposed scheme in large access hierarchies. Accordingly, the abstract has been updated in the revised manuscript.

Comment 2: It would be best to include a table summarizing the advantages and disadvantages of each research paper included in the related works to highlight what has been overcome through this study.

Response 2: We thank you for this valuable suggestion. Following your recommendation, we have added a comprehensive comparison table summarizing the main hierarchical key assignment schemes in the literature. The new Table 1 consolidates each scheme's methodology, strengths, limitations, and highlights how our proposed construction addresses the known gaps particularly regarding storage efficiency, collusion resistance, and rekeying cost.

This table is now referenced directly within the Literature Review section to enhance readability and provide a clearer contextual understanding of how our work differs from and improves upon prior approaches. We also include the table below.

Table 1: Summary of major hierarchical key assignment schemes, highlighting their methodology, advantages, limitations, and how the proposed scheme addresses their weaknesses.

**Scheme / Reference**	**Methodology / Core Technique**	**Advantages**	**Limitations / Known Weaknesses**	**How Proposed Scheme Improves**
Akl–Taylor (2)	Poset-based hierarchy; modular arithmetic	Simple; foundational model	High public storage; no temporary access; expensive updates; only KR_s	Provides basis subsets; public info independent of hierarchy size
MacKinnon et al. (7)	Canonical assignment; optimized public info	Reduced public storage	Still large storage; downward derivation; only KI_s	Subspace-based derivation prevents downward leakage
Sandhu (8)	Tree-based symmetric key; one-way function	Fast insertion; simple ops	Expensive derivation for deep trees; limited scalability	Supports arbitrary posets; basis-driven derivation avoids tree-depth cost
Harn et al. (9)	Bottom-up derivation; fewer parameters	Lower public storage	Still costly rekeying; limited flexibility	Selective subtree-only rekeying with OP mechanism
Chang–Hwang; Liaw et al. (10,11)	Newton method + one-way functions	Conceptually simple	High computational cost; collusion attacks	Resilient to collusion via basis-isolation
Liaw–Lei (13)	Up-bottom tree-based heuristic	Low storage; efficient derivation	Limited to trees; cannot handle posets	Handles general posets naturally via subspace nesting
Hwang (14)	One-way function + poset support	Supports non-tree structures; KR_s	Storage grows with poset width	Supports arbitrary width; no public info growth
Tzeng (17)	Lucas functions; time-bound keys	Time-based access; reduced public info	Computationally expensive; not KI_s under 3-user collusion	Provides time-independence; no collusion leakage
Chien et al. (18)	Tamper-resistant hardware; hash functions	High security; KR_s	Requires hardware; vulnerable to 3-user collusion	Software-only; no TTP; collusion resistance via subspace isolation
Atallah et al. (16)	Hash chains; access graphs	Dynamic; efficient; KR_s, KI_s	Storage increases with graph complexity	Fixed public vectors (f, g) independent of hierarchy
ECC-based schemes (25,26,27)	Elliptic-curve ops	Small keys; strong crypto assumptions	Some not KR_s; root-finding attacks	Independent of ECC; no algebraic attack surface
Sant–Ferara (31)	Symmetric encryption; tree heuristics	Efficient updates; KI_s	Public storage grows rapidly	No public storage; basis sets only
Ragab–Hassen (32)	Optimized symmetric approach	Low computation/storage	Still dependent on graph depth/width	Complexity independent of hierarchy shape
Najafi et al. (45)	Energy-aware; cluster-based key-server selection	Low energy use; low delay; long network lifetime	Relies on accurate position/energy data; key-server is a single point of failure	No reliance on node metrics; no single-point-failure; formal projection-based security
Cimorelli et al. (46)	PRP-based secret rotation over a plain HKAS	Avoids reinitialization; redistribution	Theoretical; No benchmarks or deployment evidence	Practical overhead analysis; no rotation dependency
Purnama et al. (47)	IOTA-integrated hierarchical IBE with delegated key derivation	Delegated; scalable access control	Not practical for real-time or large-scale IoT	No IOTA; low latency; scalable for real-time use
**Proposed Scheme**	Inner-product spaces; orthogonal projection; basis isolation	Low storage (only 2 public vectors); efficient OP-based key derivation; scalable for arbitrary posets; collusion-resistant; selective rekeying	None observed for standard threat models; numerical stability depends on implementation choice	Provides $\mathcal{O}(n_i^2)$ key derivation; no public storage growth; strong SKI_s; rekeying affects only descendants; suitable for large hierarchies

Comment 3: It would have been better to present the proposed algorithm in Figure 4 in a more detailed way, rather than just including explanatory text without any visual illustrations.

Response 3:

We sincerely thank the reviewer for highlighting the need for a clearer and more illustrative represen-

tation of the proposed algorithm. In response, we have made substantial improvements that go beyond the original request.

First, the former Figure has been fully redesigned to provide a detailed visual representation of the *System-Ready Phase*, including the system preparation (step 1a) and distribution phase (step 1b). The updated figure now clearly illustrates the initialization operations and basis distribution process, replacing the previous text-only layout with an explicit, step-wise visual diagram.

Second, to further enhance the clarity of the manuscript and to offer readers a complete view of the proposed hierarchical mechanism, we have also introduced a **new overall workflow diagram**. This new figure combines all major phases of the scheme (the System-Ready Phase, the Key Derivation Phase, and the Dynamic Update / Rekeying Phase) into a unified end-to-end visual workflow. It summarises the complete process described in Algorithm 1 and Algorithm 2 and provides a comprehensive overview of how the system operates in practice.

Together, these two figures provide both a phase-specific and system-level perspective, significantly improving the readability and completeness of the methodological presentation. The caption and relevant discussion in the manuscript have been updated accordingly.

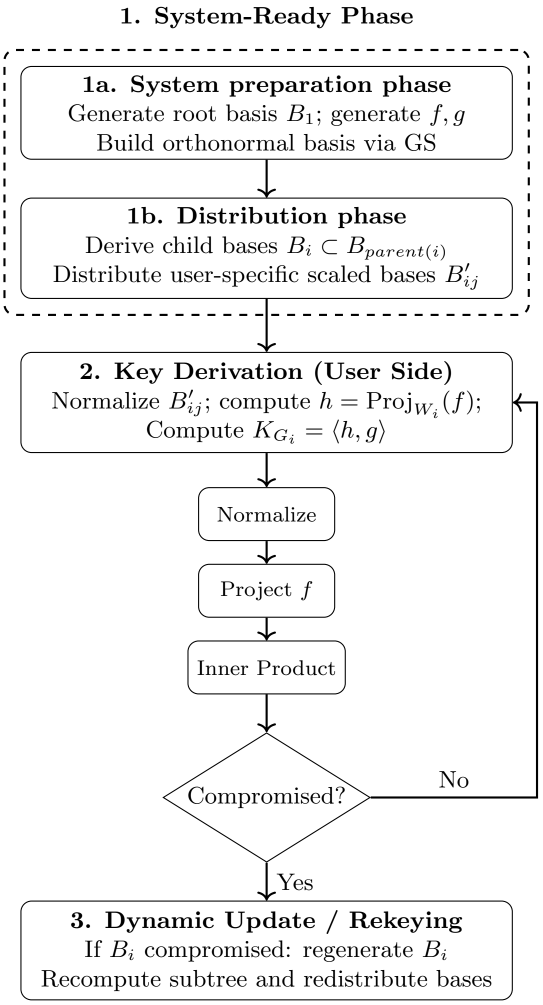

Figure 2: Workflow diagram for the proposed hierarchical key assignment scheme. Steps 1a and 1b are grouped under the System-Ready Phase, followed by key derivation and dynamic rekeying with compromise detection.

Comment 4: A large proportion of the references were published before 2020, so it is preferable to cover all of the last five years, especially the period between 2023-2025, with no less than eight research papers within the last three years.

Response 4: We sincerely appreciate your valuable suggestion. In response to your comment, we have expanded the literature review to incorporate several recent works published between 2023 and 2024. Specifically, five new studies (45–49) have been added to the revised manuscript, and the discussion

has been updated to reflect contemporary advances in hierarchical key management and access-control systems. With these additions, the related work section now includes more than eight recent publications.

Response to Reviewer #3

Comment 1: Add more recently published works.

Response 1: We sincerely appreciate your helpful suggestion. In the revised manuscript, we have expanded the related-work section to include additional contributions published in 2023 and 2024. Specifically, five new studies (45–49) addressing hierarchical key management and access-control mechanisms have been incorporated, and the corresponding discussion in the literature review has been refined to clarify how these contemporary approaches relate to our construction.

Comment 2: Clearly present your contributions compared with recent related works in table form.

Response 2: We sincerely appreciate your constructive suggestion. In accordance with the recommendation, we have added a new comparative table (Table 1) that systematically contrasts the most influential hierarchical key assignment schemes with our proposed construction. The table includes a dedicated column explaining how our scheme improves upon each prior work, highlighting advances in collusion resistance, reduced storage overhead, independence from graph depth and width, and efficient selective rekeying via orthogonal projection. All compared works, including recent contributions, are summarized in terms of methodology, advantages, known weaknesses, and the specific improvements introduced by our inner-product–based hierarchical mechanism. We are hoping that this addition now prodive a clear and comprehensive overview of how our contributions address longstanding challenges in hierarchical key assignment.

Comment 3: Explain how the system detects if a basis set has been compromised.

Response 3: We thank you for raising the important question regarding how the system proceeds in the event that a basis is compromised. In the revised manuscript, we have clarified that the detection of compromised group bases must occur at the operational layer, since the cryptographic scheme itself does not possess the inherent capability to autonomously determine compromise. If a basis is exposed, an adversary can trivially derive the corresponding group key; therefore, detection must rely on external indicators such as anomalous key derivations, irregular access attempts, or systematic monitoring of access logs.

To address this concern, we have explicitly described the recovery procedure: once compromise is confirmed, the selective rekeying mechanism outlined in Algorithm 2 is initiated. This mechanism recomputes and securely redistributes only the affected subtree, thereby minimizing communication overhead while preserving the integrity and continuity of the remaining hierarchy. By restricting rekeying to the compromised branch, the scheme ensures that unaffected portions of the hierarchy remain valid, which significantly reduces operational disruption compared to global rekeying approaches. To address this concern, we have added Remark 3, explicitly indicating how the system should proceed in the case of a compromised basis.

Comment 4: Add comprehensive benchmarks including memory usage and network bandwidth for key distribution.

Response 4: We thank you for this valuable suggestion. In accordance with the recommendation, we have added a new benchmark subsection that provides quantitative measurements for both the memory usage and the communication bandwidth required during the key distribution phase.

Specifically, we now present the exact bit-level storage size of the scaled basis B'_{ij} that is delivered to each user, derived as $n_i m\lambda$ bits, and we provide numerical evaluations for the system parameters used in our experiments ($m = 32$, $n_i = 10$, $\lambda = 32$), showing that each user receives only 10,240 bits (≈1.25 KB) of key material. This demonstrates the compactness and suitability of the scheme for resource-constrained environments.

Furthermore, we include a detailed bandwidth analysis for both initial key distribution and selective rekeying. Since the communication cost of key distribution equals the size of the transmitted basis, the bandwidth per user is also 10,240 bits. For rekeying, the communication overhead is shown to scale linearly with the number of affected users m', requiring $m' \cdot n_i m\lambda$ bits in total.

All results are summarized in a new table added to the manuscript (see the section highlighted in blue). These benchmarks complement the theoretical comparison already provided in Table 3 and offer a clear quantitative evaluation of the practical efficiency of the proposed scheme.

Comment 5: Add a concrete security analysis section with parameter recommendations aligned to NIST security levels.

Response 5: We thank you for highlighting the importance of security parameterization in the context of NIST post-quantum standards. In the revised manuscript, we have added a dedicated subsection that provides explicit parameter recommendations aligned with the NIST PQC security categories (Levels 1, 3, and 5), following the guidelines in NIST SP 800-57. This subsection specifies how the security of our scheme depends on the universal space dimension m, the dimension n_i of each subspace W_i, and the bit-length λ of the basis coordinates, and it presents recommended values for each parameter set to achieve 128-bit, 192-bit, and 256-bit classical security. We also emphasize that the scheme can be optionally hybridized with NIST-recommended post-quantum KEMs (e.g., CRYSTALS-Kyber), thereby providing an additional confidentiality layer for applications requiring formal PQC assurances or regulatory compliance. We believe this addition directly addresses your request by offering clear, standards-aligned guidance for parameter selection and demonstrating the modularity of our design in both classical and post-quantum environments.
