# Supplementary material for: Hierarchical secure key assignment scheme
Source: PLoS One. 2026 Feb 18;21(2):e0341637. doi: 10.1371/journal.pone.0341637 (PMC12915986; doi:10.1371/journal.pone.0341637)
Supplement: Attachment — Submitted filename: Comments for the Author.docx [file pone.0341637.s002.docx]

Comments to the Author

A study presented novel ideas based on orthogonal projection and distributing a basis. However paper should be accepted after minor revisions and corrected the following issues.

1.Add more recently published works

2.Clearly present your contributions compared with recent related works in table form.

3.Explain how does the system detect if a basis set has been compromised?

4. Add comprehensive benchmarks including memory usage, network bandwidth for key distribution.

5.Add a concrete security analysis section with parameter recommendations aligned to NIST security levels.
